# The Role of Estrogen Membrane Receptor (G Protein-Coupled Estrogen Receptor 1) in Skin Inflammation Induced by Systemic Lupus Erythematosus Serum IgG

**DOI:** 10.3389/fimmu.2017.01723

**Published:** 2017-12-04

**Authors:** Zhenming Cai, Changhao Xie, Wei Qiao, Xibin Fei, Xuanxuan Guo, Huicheng Liu, Xiaoyan Li, Xiang Fang, Guangqiong Xu, Hui Dou, Guo-Min Deng

**Affiliations:** ^1^Key Laboratory of Antibody Techniques of Ministry of Health, Nanjing Medical University, Nanjing, China; ^2^State Key Laboratory of Reproductive Medicine, Nanjing Medical University, Nanjing, China; ^3^First Affiliated Hospital, Nanjing Medical University, Nanjing, China

**Keywords:** estrogen, systemic lupus erythematosus, IgG, skin inflammation, G protein-coupled estrogen receptor 1

## Abstract

Skin injury is the second most common clinical manifestation in patients with systemic lupus erythematosus (SLE). Estrogen may affect the onset and development of SLE through its receptor. In this study, we investigated the role of estrogen membrane receptor G protein-coupled estrogen receptor 1 (GPER1) in skin injury of SLE. We found that skin injury induced by SLE serum was more severe in female mice and required monocytes. Estrogen promoted activation of monocytes induced by lupus IgG through the membrane receptor GPER1 which was located in lipid rafts. Blockade of GPER1 and lipid rafts reduced skin inflammation induced by SLE serum. The results we obtained suggest that GPER1 plays an important role in the pathogenesis of skin inflammation induced by lupus IgG and might be a therapeutic target in skin lesions of patients with SLE.

## Introduction

Systemic lupus erythematosus (SLE) is a chronic autoimmune disease characterized by high levels of autoantibodies and multi-organ tissue damage (including the kidney, skin, lungs, brain, and heart) (1, 2). The prevalence of SLE in population ranges from 20 to 150 cases per 100,000, with up to 90% affecting women of childbearing age (3, 4). The female-to-male ratio in prepuberty is 3:1, and this ratio increases to 10:1 during the reproductive age and decreases again to 8:1 after menopause (5). In SLE patients, serum estrogen levels are much higher than in healthy subjects and correlated with disease severity (6, 7). Using of exogenous estrogen could increase the risk of SLE development in healthy women and exacerbate the disease in SLE patients and mouse models (8–10). It was reported that in (NZB × NZW)F1 mice, lupus development was strongly influenced by gender, and lupus incidence was higher, and survival time was reduced in female mice compared with males (11). In addition, exogenous estrogens also could accelerate lupus development and autoantibody production in male and female (NZB × NZW)F1 mice (12, 13). These data suggest that estrogen may have an important role in the onset and development of SLE.

Estrogens have important influences on several immune functions (14). It has been shown that female sex hormones could affect T cell function and antibody production in B cells. Estrogens are a class of sex steroid hormones that were synthesized from cholesterol. Three forms of physiological estrogens exist in female animals: estrone (E1), estradiol (E2, or 17-β estradiol), and estriol (E3). E2 is the staple product of this process and the most potent estrogen during the childbearing age (15). Estrogen binds two types of receptors, which were named nuclear receptors (ERα and ERβ) and cell membrane receptors [G protein-coupled estrogen receptor 1 (GPER1) and ER-X], to trigger direct and indirect responses within the cell (16). ERα and ERβ which belong to the superfamily of nuclear receptors are soluble receptors that shuttle between the cytoplasm and the nucleus (17). After binding estrogen-responsive elements (EREs) and recruiting co-regulatory and chromatin remodeling proteins, ERα or ERβ regulates transcription of target genes (18). GPR30 which is now named GPER1 is a seven transmembrane-domain G protein-coupled receptor. As an integral membrane estrogen receptor, GPER1 can elicit rapid estrogen-responsive signaling independent of ERα and ERβ (19).

It has been shown that mortality and glomerulonephritis severity were significantly decreased in ERα-deficient female (NZB × NZW)F1 mice (20), and ERα activation has an immunostimulatory role in murine lupus, whereas ERβ activation performs mild immunosuppressive effects (21). In human studies, it has been found that polymorphisms of ERα but not ERβ may be associated with susceptibility or development of SLE (22, 23). These data suggest that ERα plays a main role in mediating the effects of estrogens in SLE. However, the role of estrogen membrane receptor GPER1 in the onset and development of SLE remains unclear.

In the patients with SLE, skin injury is the second most common clinical manifestation (24). However, the role of estrogen in the development of skin inflammation is not well known. In this study, we investigated the role and mechanism of estrogen in the development of skin inflammation in SLE. We found that estrogen could promote the development of skin injury induced by SLE serum through GPER1 and that lipid rafts serve an important function in the regulatory effect of GPER1 in skin inflammation induced by SLE IgG.

## Materials and Methods

### Information of SLE Patients

A total of 398 SLE patients hospitalized in The First Affiliated Hospital of Bengbu Medical College between January 2010 and December 2011 were recruited. All patients fulfilled at least four of the American College of Rheumatology criteria for SLE (25). The local ethics committee approved the protocol and written informed consents were obtained.

### Mice and Reagents

The Csf-1-deficient mice were purchased from The Jackson Laboratory, and C57BL/6 mice were purchased from Nanjing University. All mice were housed in the animal facility of Nanjing Medical University.

Sera of SLE patients were collected from The First Affiliated Hospital of Nanjing Medical University. We adopt the criteria of the American College of Rheumatology as the classification of SLE. All patients had SLE disease-activity index scores ranging from 0 to 20.

Protein G agarose was purchased from Millipore. FITC-conjugated CTB, methyl-β-cyclodextrin (MβCD) and β-estradiol 6-(*O*-carboxy-methyl)oxime: BSA (E2-BSA) were purchased from Sigma-Aldrich. G15 was purchased from TOCRIS bioscience. ICI 182,780 (Fulvestrant), a specific antagonist of nuclear estrogen receptors, was purchased from Santa Cruz Biotechnology.

Anti-CD11b, TNF-α, and MCP-1 antibodies were purchased from Abcam. Anti-phospho-NF-kB p65 rabbit mAb, NF-κB p65 rabbit mAb, and GAPDH rabbit mAb antibodies were purchased from Cell Signaling Technology. Anti-CD3, CD20, GPER1, and CD64 antibodies were purchased from Santa Cruz Biotechnology.

### Injection Protocol

Systemic lupus erythematosus serum (100 µl) or SLE IgG (50 μg/100 μl) was injected intradermally in the back of the neck of treated mice and PBS (100 µl) was used as control. MβCD (1 mg/mouse, in a volume of 100 µl) or G15 (2 μg/mouse, in a volume of 100 µl) were intraperitoneally injected three times a week for 2 weeks before SLE serum injection. Control mice received the same volume of the PBS.

### Histopathological and Immunohistochemical Examination of Skin Lesions

Histopathological examination of skin tissue was performed as described earlier (26). After routine fixation and paraffin embedding, tissue sections from skin were cut and stained with H&E. The slides were coded and evaluated in a blinded manner. Skin inflammation severity was scored as described before (26).

After deparaffinization and Ag retrieval, the skin tissues were stained with antibodies to CD3, CD11b, CD20, α-NAE, MCP-1, and TNF-α followed by incubation with biotinylated secondary antibodies, avidin–biotin–peroxidase complexes, and 3-amino-9-ethyl-carbazole-containing H_2_O_2_. All sections were counterstained with Mayer’s hematoxylin (26).

### Monocyte Differentiation into Dendritic Cells (DCs)

Mononuclear cells isolated from spleens of C57BL/6 mice were incubated in 24-well plates at 37°C for 3 h and then monocytes were obtained after removing suspended lymphocytes. Monocytes were cultured in DMEM medium with 5% FBS, on 37°C, and 5% CO_2_. Monocytes were stimulated with SLE serum or SLE IgG for 3 and 48 h. Then, we examined the differentiation of monocytes into DCs by light microscopy. RAW264.7 cells were cultured in DMEM medium with 5% FBS, on 37°C, and 5% CO_2_.

### Measurement of MCP-1 and TNF-α

The levels of MCP-1 and TNF-α in supernatants were determined by using a mouse MCP-1 Flex cytokine kit, mouse TNF-α Flex cytokine kit and cytometric bead array according to the manufacturer’s instructions (BD Biosciences).

### Western Blotting and Coimmunoprecipitation

Raw264.7 cells were lysed in RIPA buffer, and the supernatants of homogenates were subjected to SDS-PAGE. Blotted onto polyvinylidene difluoride (PVDF) membranes and stained for p-NF-κB p65/NF-κB p65 and GAPDH.

Raw264.7 cells were lysed in RIPA buffer, and homogenates were incubated with protein G beads pre-absorbed with anti-CD64 antibody. Pre- and post-CD64-immunoprecipitated supernatants and anti-CD64 beads were subjected to SDS-PAGE, blotted onto PVDF membranes, and stained for GPER1.

### Immunofluorescent Staining

After fixing with 3% paraformaldehyde in PBS and cytospuning onto slides, monocytes were permeabilized with 0.05% Triton X-100 for 5 min at room temperature and blocked with PBS containing 10% normal goat serum for 1 h. Then, the cells were incubated with primary antibodies (CD64 or GPER1) in 0.5% BSA at room temperature for 45 min. The cells were washed after incubating with a secondary antibody and FITC-conjugated CTB for 45 min; the slides were washed three times by PBS. After staining the nuclei with DAPI for 5 min, the slides were mounted with a coverslip using Fluoromount-G, and cells were observed using a Zeiss LSM 510 META confocal microscope (26).

### Statistics

Statistical evaluations of skin inflammation severity, levels of MCP-1 and TNF-α were performed using Student’s *t*-test. *P* < 0.05 was considered statistically significant.

## Results

### Estrogen Is Involved in the Pathogenesis of SLE

Because females have high estrogen levels, we investigated female SLE patients to determine the role of estrogen in the pathogenesis of SLE. We investigated 398 SLE patients and found that 374 (94.0%) of the patients were females and 279 (83.7%) were in the childbearing age (18–45 years) (Table 1). These data suggest that estrogen may play a role in the pathogenesis of SLE.

**Table 1:** The demographics of systemic lupus erythematosus patients.

Sex		
	Female	Male
	374	24
Age (years)		
Median	34.5 ± 13.4	31.8 ± 13.9
Range	10–69	13–76
<18	23 (6.1%)	2
18–45	313 (83.7%)	20
>45	38 (10.2%)	2

**Figure 1:**
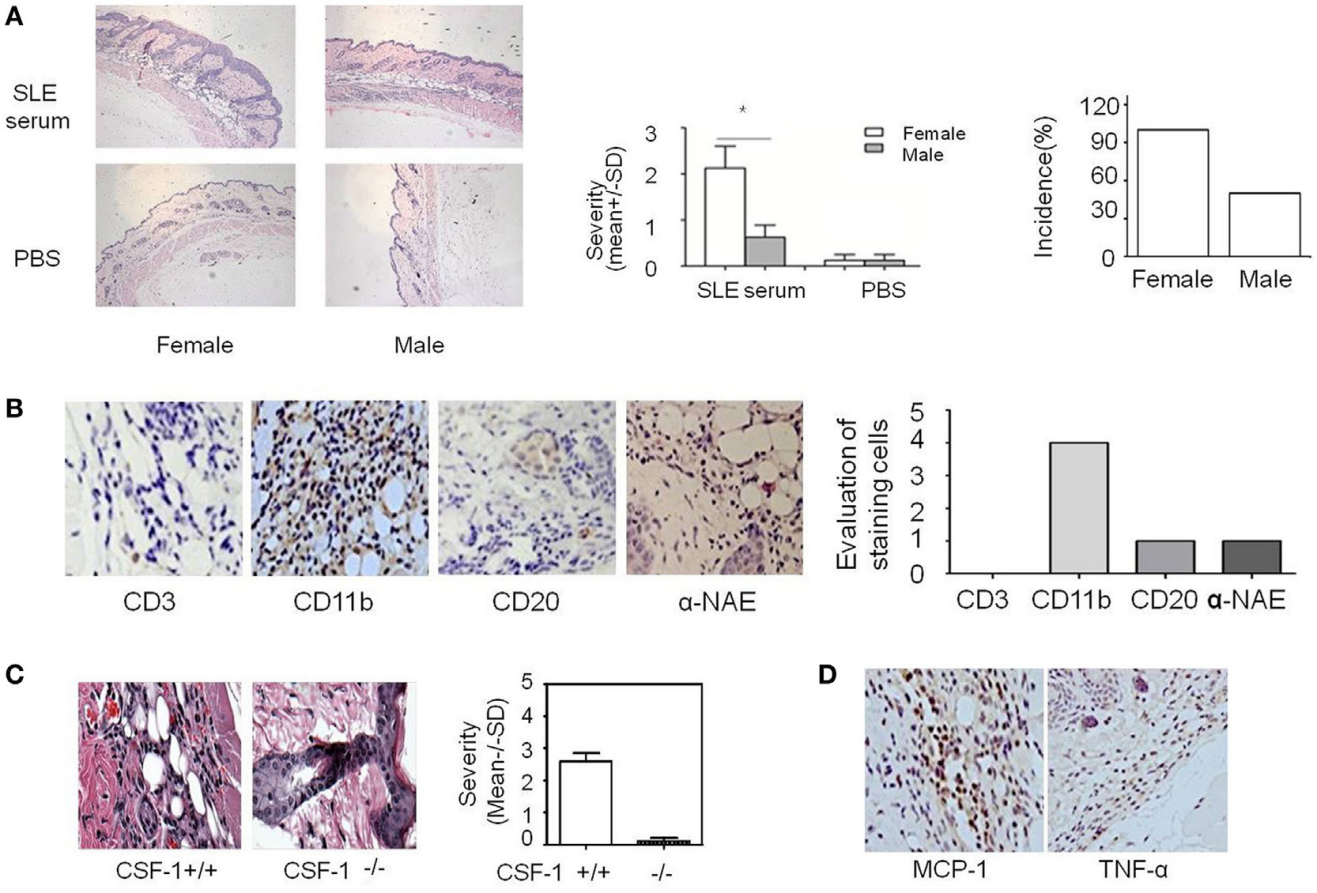
Analysis of systemic lupus erythematosus (SLE) serum-induced skin inflammation in mice and the role of monocytes in this progress. **(A)** Representative histopathological photomicrograph of the severity and incidence of skin inflammation in female and male C57BL/6 mice sacrificed 3 days after intradermal inoculation of serum (100 µl) from SLE patients (*n* = 8 per group). H&E, original magnification 20×. **(B)** Immunohistochemical staining for CD3, CD11b, CD20, and α-NAE expression in skin lesions of C57BL/6 mice 3 days after intradermal inoculation of serum (100 µl) from SLE patients (the scoring system: 0, no positive cells; 1, 1–5 positive cells; 2, 6–10 positive cells; 3, 10–20 positive cells; 4, >20 positive cells in a field). **(C)** Severity and picture of SLE serum-induced skin inflammation in female Csf-1^−/−^ mice and wild-type mice (*n* = 5 per group) 3 days after intradermal inoculation of serum (100 µl) from SLE patients. **(D)** Immunohistochemical staining for MCP-1 and TNF-α expression in skin lesions of C57BL/6 mice 3 days after intradermal inoculation of SLE serum. *P < 0.05.

To further investigate whether estrogen regulates SLE-related organ and tissue damage, we used an animal model in which SLE serum is administered to induce skin inflammation (26). We injected SLE serum intradermally in female and male C57BL/6 mice. Histopathological examination demonstrated that resulting skin lesions were more severe in female mice than in males (Figure 1A). These data suggest that female mice are more susceptible to SLE serum-induced skin inflammation and estrogen may be involved in the pathogenesis of skin inflammation in SLE. We also use serum from healthy volunteer as negative controls and we did not find skin inflammation induced by healthy volunteer and PBS (Figure S1 in Supplementary Material).

### Monocytes Contribute to the Development of SLE Serum-Induced Skin Inflammation

Because estrogen may regulate the severity of SLE serum-induced skin inflammation, we investigated the role of its immune cell targets in this regulatory effect. Immunohistochemical staining demonstrated that there were abundant CD11b^+^ cells but very few CD3^+^, CD20^+^, and α-NAE^+^ cells in skin lesions (Figure 1B). These data indicate that monocytes/macrophages may play an important role in the development of SLE serum-induced skin inflammation. Previous work has shown that monocytes/macrophages, but not lymphocytes, were important in the development of this inflammation (26). To further verify the role of monocytes/macrophages in SLE serum-induced skin inflammation, we tried to induce skin inflammation in Csf-1^−/−^ mice lacking mature monocytes. Compared to wild-type mice, we found that the severity and incidence of skin inflammation decreased significantly in Csf-1^−/−^ mice (Figure 1C). In addition, the expression of MCP-1 and TNF-α was increased in skin lesions of mice (Figure 1D). These data suggest that monocytes may play an important role in the development of SLE serum-induced skin inflammation.

### Lipid Rafts Have an Important Regulatory Role in the Activity of Monocytes Triggered by SLE IgG

**Figure 2:**
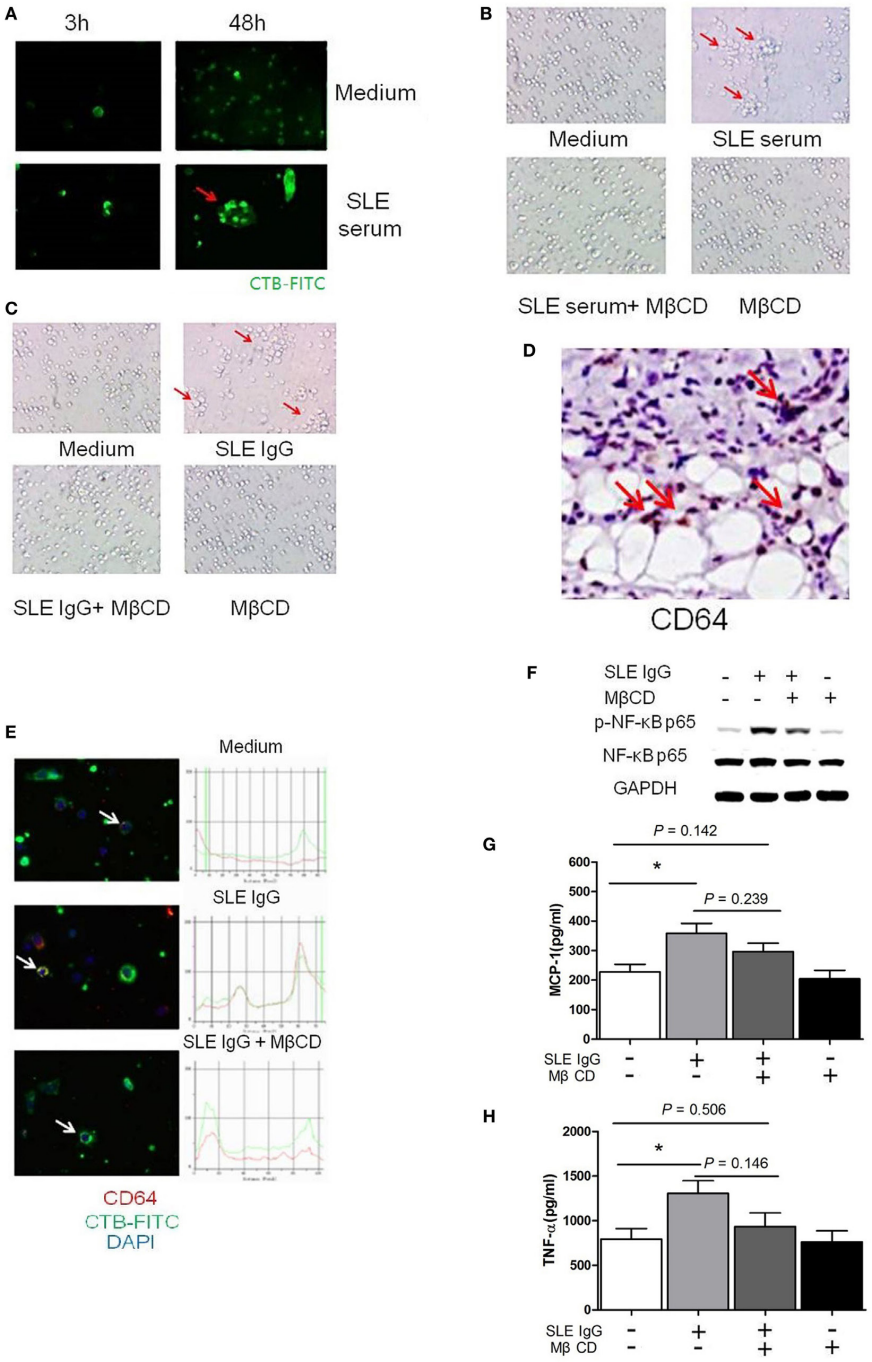
Lipid rafts are important in the process of systemic lupus erythematosus (SLE) IgG-induced differentiation of monocytes into dendritic cells (DCs). **(A)** Confocal microscopy analysis of lipid raft clustering using FITC-conjugated CTB in monocytes cultured with SLE serum or medium control for 3 and 48 h. The arrows represent typical clustered lipid rafts. **(B,C)** Representative photographs of monocytes cultured with SLE serum with or without methyl-β-cyclodextrin (MβCD) for 48 h **(B)** or SLE IgG with or without MβCD for 48 h **(C)**. Original magnification 20× and the arrows represent typical differentiation of monocytes into DCs. **(D)** Immunohistochemical staining for CD64 (FcγRI) expression in skin lesions of C57BL/6 mice 3 days after intradermal inoculation of SLE serum. The arrows represent CD64^+^ cells. **(E)** Confocal microscopy analysis of colocalization of CD64 and lipid rafts in monocytes cultured with medium control, SLE IgG, SLE IgG, and MβCD for 3 h. The arrows represent the cells which were analyzed. **(F)** Western blot analysis of expression of p-NF-κB p65/NF-κB p65 and GAPDH in Raw264.7 cells treated with medium control, SLE IgG, SLE IgG + MβCD, or MβCD for 30 min. **(G,H)** CBA-measured levels of MCP-1 or TNFα in supernatants of Raw264.7 cells treated with medium control, SLE IgG, SLE IgG + MβCD, or MβCD for 24 h. *P < 0.05.

Lipid rafts are sphingolipid- and cholesterol-rich domains of the plasma membrane that contain various signaling and transport proteins (27). These domains contribute significantly to the pathogenesis of organ and tissue damage in lupus-prone mice (28). Based on this information, we next investigated whether SLE serum triggered lipid raft aggregation on monocytes. We found that clustering of lipid rafts developed on monocytes 3 and 48 h after SLE serum treatment (Figure 2A). These data suggest that SLE serum-induced lipid raft clustering on monocytes.

Because SLE serum can induce differentiation of monocytes into DCs (26), we next investigated whether depletion of lipid rafts using MβCD would affect this differentiation. We found that lipid raft depletion inhibited both SLE serum-induced and SLE IgG-induced monocyte differentiation into DCs (Figures 2B,C). These data indicate that lipid rafts have an important role in monocyte differentiation into DCs.

To understand how lipid rafts regulate the effects of SLE IgG which is major contributor in SLE serum-induced skin inflammation, we assessed whether they contained Fcγ receptors (FcγRs) which are IgG receptors (29). First, we analyzed the expression of FcγRI (CD64) in skin lesions induced by SLE serum and found a large amount of CD64^+^ cells (Figure 2D). Then, we analyzed whether lipid rafts contain CD64. We observed colocalization of CD64 and lipid rafts in monocytes treated with SLE IgG and this colocalization was abolished by MβCD treatment (Figure 2E).

To further characterize the regulatory role of lipid rafts in the expression of intracellular signaling molecules induced by SLE IgG, we analyzed the expression of p-NF-κB p65/NF-κB p65 in Raw264.7 cells. We found that SLE IgG increased levels of p-NF-κB p65, MCP-1, and TNF-α in supernatants of Raw264.7 cells (Figures 2F–H). Conversely, MβCD decreased the expression of p-NF-κB p65, MCP-1, and TNF-α. These results indicate that lipid rafts are important in the expression of inflammatory and intracellular signaling molecules in monocytes triggered by SLE IgG.

### E2 Promotes Activation of SLE IgG-Induced Monocytes *via* the Membrane Receptor GPER1

Estrogens exert their functions by binding nuclear receptors (ERα and ERβ) that act as ligand-dependent transcription factors or by binding membrane-bound receptors (GPR30 and ER-X) that initiate signal transduction pathways (16). Although several studies have shown that ERα plays a major role in mediating the effects of estrogens in SLE (20, 21), there are no data to describe the role of the estrogen cell membrane receptor GPER1 (also called GPR30) in SLE. To determine the role of GPER1 in SLE, we used E2-BSA, an activator specifically bound to GPER1.

**Figure 3:**
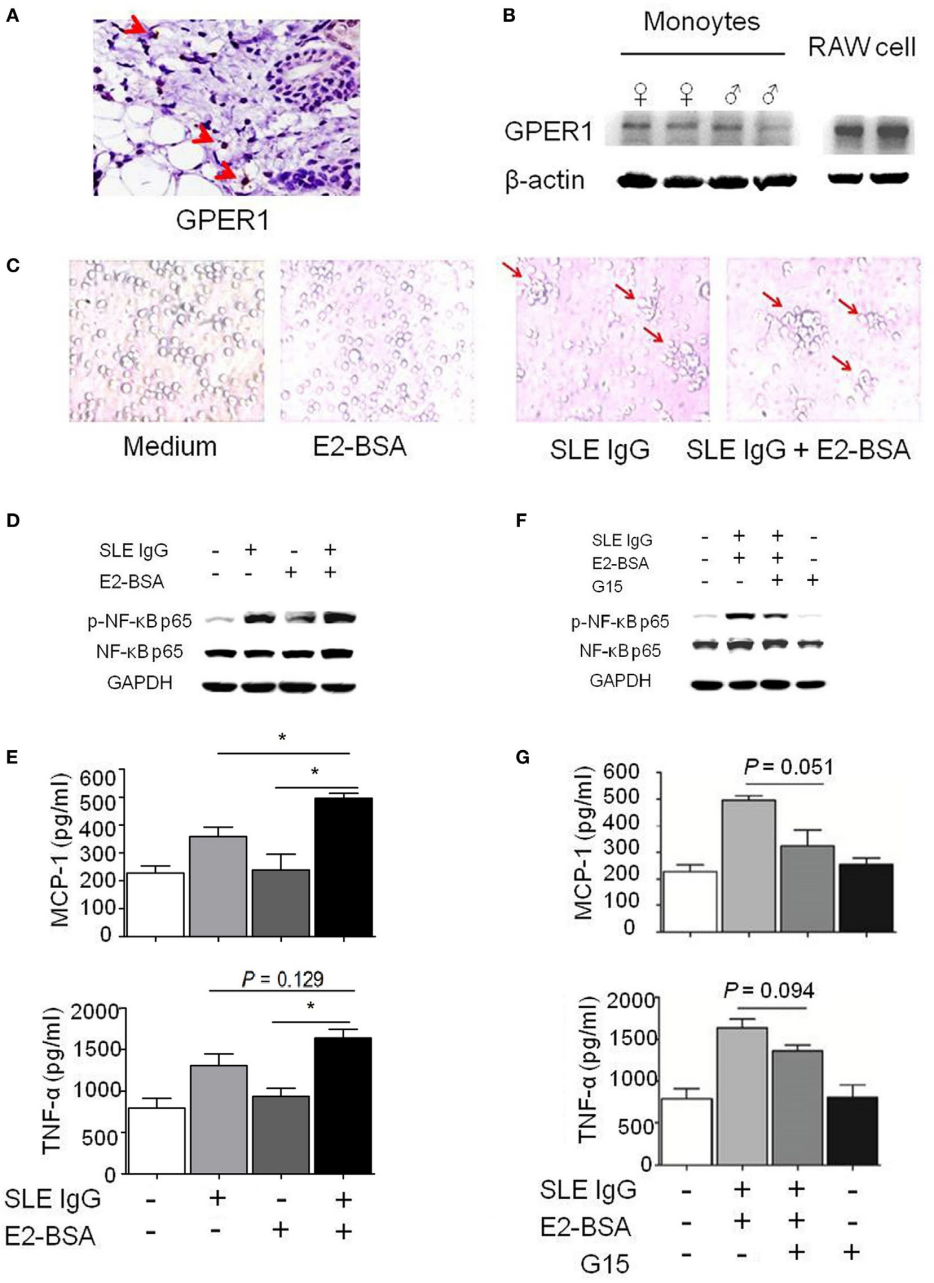
E2 promotes activation of systemic lupus erythematosus (SLE) IgG-induced monocytes *via* the membrane receptor G protein-coupled estrogen receptor 1 (GPER1). **(A)** Immunohistochemical staining for GPER1 expression in skin lesions of C57BL/6 mice 3 days after intradermal inoculation of SLE serum. The arrows represent GPER1^+^ cells. **(B)** Western blot-measured GPER1 expression in Raw264.7 cells and monocytes from C57BL/6 mice. **(C)** Representative photographs of monocytes cultured with medium control, E2-BSA, SLE IgG, or SLE IgG + E2-BSA for 48 h. Monocytes were isolated from spleen of C57BL/6 mice. Original magnification 20× and the arrows represent typical differentiation of monocytes into dendritic cells. **(D)** Western blot-measured levels of p-NF-κB p65/NF-κB p65 in Raw264.7 cells treated with medium control, SLE IgG, E2-BSA, or SLE IgG + E2-BSA for 30 min. **(E)** CBA-measured levels MCP-1 or TNF-α in supernatants of Raw264.7 cells treated with medium control, SLE IgG, E2-BSA, or SLE IgG + E2-BSA for 24 h. *P < 0.05. **(F)** Western blot-measured levels of p-NF-κB p65/NF-κB p65 in Raw264.7 cells treated with medium control, SLE IgG + E2-BSA, SLE IgG + E2-BSA + G15, or G15 for 30 min. **(G)** CBA-measured levels of MCP-1 or TNF-α in supernatants of Raw264.7 cells treated with medium control, SLE IgG + E2-BSA, SLE IgG + E2-BSA + G15, or G15 for 24 h.

First, we investigated whether monocytes express GPER1. We found a number of GPER1^+^ cells in the skin lesions induced by SLE serum (Figure 3A). We also saw GPER1 expression in monocytes from C57BL/6 mice and Raw264.7 cells by western blotting, and there was no significant difference of GPER1 expression between female and male mice (Figure 3B). Next, we determined whether E2-BSA regulates the activity of monocytes triggered by SLE IgG. We observed that SLE IgG or SLE IgG with E2-BSA could induce monocyte differentiation into DCs but not E2-BSA alone (Figure 3C). There were no significant differences between cells treated with SLE IgG or SLE IgG with E2-BSA. These data indicate that E2-BSA alone did not enhance SLE IgG-induced monocyte differentiation into DCs.

To investigate the effect of E2-BSA on expression of intracellular signaling molecules induced by SLE IgG, we analyzed the expression of p-NF-κB p65/NF-κB p65 in Raw264.7 cells treated with SLE IgG in the presence or absence of E2-BSA. We found that E2-BSA increased levels of p-NF-κB p65 induced by SLE IgG compared with the cells treated with SLE IgG only (Figure 3D). We also measured the levels of MCP-1 and TNF-α in supernatants of Raw264.7 cells treated with SLE IgG in presence or absence of E2-BSA for 24 h and found that E2-BSA increased levels of MCP-1 and TNF-α triggered by SLE IgG (Figure 3E). To further confirm the effect of estrogen membrane receptor on SLE IgG, we used G15, a specific antagonist of GPER1 (30). We found that G15 abolished the effect of E2-BSA on expression of p-NF-κB p65 (Figure 3F) and expression of MCP-1 and TNF-α triggered by SLE IgG (Figure 3G).

To further determine whether estrogen nuclear receptors regulate activation of NF-κB p65 triggered by SLE IgG, we blocked the effect of ERα and ERβ by using ICI 182,780 (Fulvestrant), a specific antagonist of nuclear estrogen receptors. We found that blockade of estrogen nuclear receptors did not inhibit the effect of E2-BSA on activation of NF-κB p65 triggered by SLE IgG (Figure S2A in Supplementary Material).

In addition, we further investigated the effects of estrogen and G15 on activation of NF-κB p65 triggered by SLE IgG. We found that E2, E2-BSA, or G15 did not directly activate NF-κB p65; E2-BSA enhanced but G15 decreased activation of NF-κB p65 triggered by SLE IgG; E2 did not affect levels of p-NF-κB p65 induced by SLE IgG (Figure S2B in Supplementary Material). These results indicate that estrogen membrane receptor not nuclear receptors promote activation of NF-κB p65 triggered by SLE IgG.

To determine whether GPER1 activation enhances skin inflammation induced by SLE serum, we treated mice with intradermal injection of SLE serum in the presence or absence of E2-BSA. As shown in Figure S3 in Supplementary Material, E2-BSA alone did not induce skin inflammation, but enhanced the skin inflammation induced by SLE serum. These data suggest that activated membrane receptor of estrogen may promote skin inflammation induced by SLE serum.

### E2 Promotes Activation of SLE IgG-Induced Monocytes through Lipid Rafts

**Figure 4:**
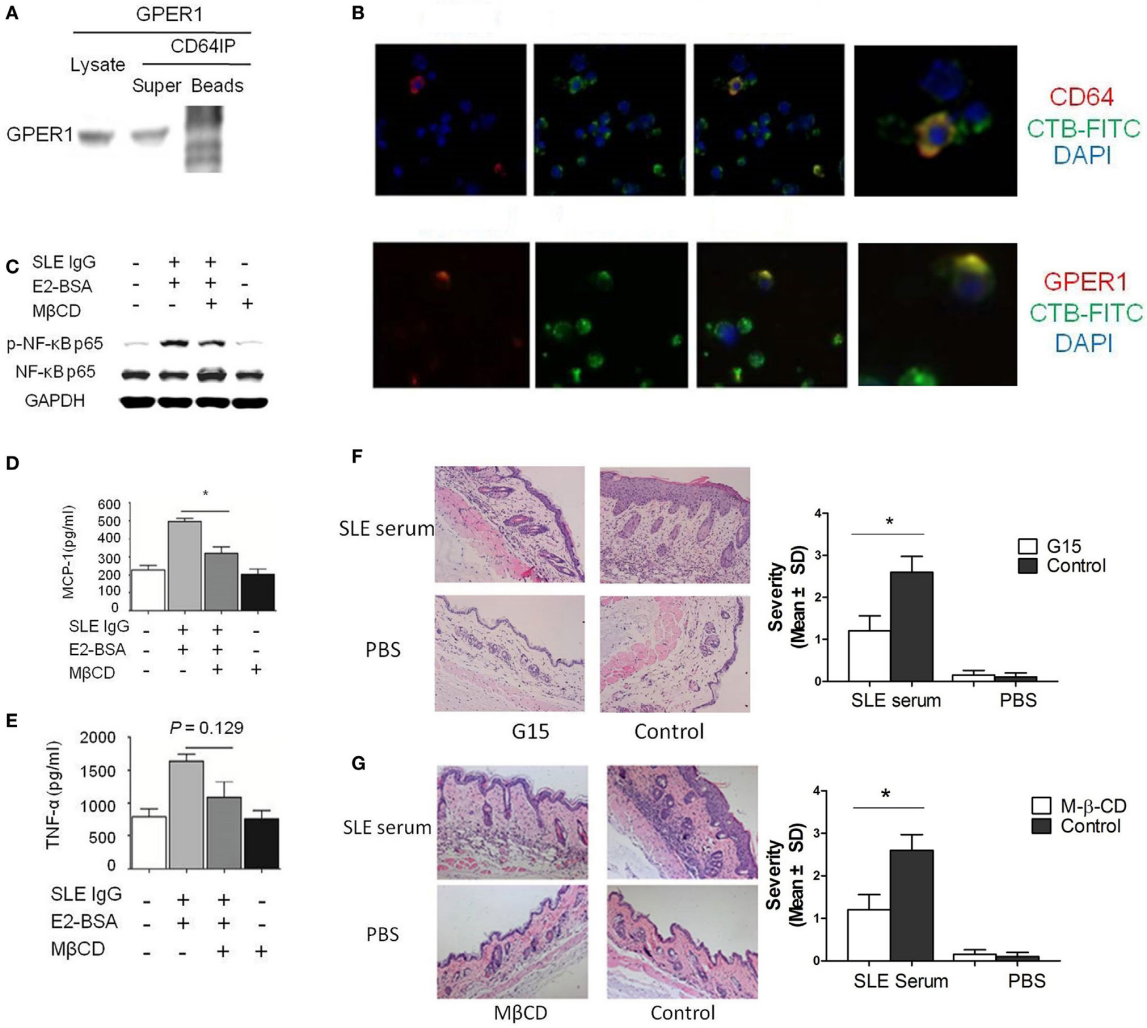
E2 promotes activation of systemic lupus erythematosus (SLE) IgG-induced monocytes and skin inflammation through lipid rafts. **(A)** Relationship of G protein-coupled estrogen receptor 1 (GPER1) and CD64 measured by western blot and immunoprecipitation in Raw264.7 cells. **(B)** Immunofluorescent staining of GPER1 and lipid rafts or CD64 and lipid rafts on monocytes treated with SLE IgG. **(C)** Western blot-measured levels of p-NF-κB p65/NF-κB p65 in Raw264.7 cells treated with medium, SLE IgG + E2-BSA, SLE IgG + E2-BSA + methyl-β-cyclodextrin (MβCD), or MβCD for 30 min. **(D,E)** CBA-measured levels of MCP-1 **(D)** or TNF-α **(E)** in supernatants of Raw264.7 cells treated with medium, SLE IgG + E2-BSA, SLE IgG + E2-BSA + MβCD, or MβCD for 24 h. **(F)** Histopathological photomicrograph and severity of skin inflammation in female C57BL/6 mice treated with or without G15 and sacrificed 3 days after intradermal inoculation of SLE serum. G15 at dose of 2 µg was administered intraperitoneally three times a week for 2 weeks (*n* = 10 per group). **(G)** Histopathological photomicrograph and severity of skin inflammation in female C57BL/6 mice treated with or without MβCD (1 mg/mouse three times a week, i.p., for 2 weeks) sacrificed 3 days after intradermal inoculation of serum (100 µl) from a SLE patient (*n* = 8 per group). H&E, original magnification 20×. *P < 0.05.

E2 promotes SLE IgG-induced monocyte activation and cytokine production *via* GPER1 but the mechanism was still unknown. We hypothesized that GPER1 might bind to CD64 to promote this process. We used coimmunoprecipitation to identify the relationship between GPER1 and CD64. As shown in Figure 4A, we did not find direct binding of GPER1 and CD64.

Because GPER1 and CD64 are membrane receptors and we have shown that CD64 localized in clustered lipid rafts in SLE serum-treated monocytes, we investigated whether clustered lipid rafts contain GPER1 and CD64. We found that GPER1 and CD64 colocalized with clustered lipid rafts in SLE serum-treated monocytes (Figure 4B). These results indicate that lipid rafts serve as platforms for the interaction between GPER1 and CD64. To confirm this point, we used MβCD to inhibit the lipid raft clustering in Raw264.7 cells treated with SLE IgG in the presence or absence of E2-BSA. We found that MβCD decreased levels of p-NF-κB p65 trigged by SLE IgG with E2-BSA (Figure 4C). We also found that MβCD inhibited the effect of E2-BSA on SLE IgG-induced expression of MCP-1 and TNF-α (Figures 4D,E). These results indicated that E2 promoted SLE IgG-induced monocyte activation through lipid rafts.

### Inhibition of GPER1 and Lipid Rafts Reduced SLE Serum-Induced Skin Inflammation

We have shown that G15 and MβCD could inhibit SLE IgG-induced monocyte activation and cytokine production. Here, we investigated the effect of G15 and MβCD in SLE serum-induced skin inflammation. We found that the severity of SLE serum-induced skin inflammation was significantly decreased in the mice treated with G15 (Figure 4F) and mice treated with MβCD (Figure 4G). In addition, we analyzed the serum E2 levels of female mice treated with MβCD or G15. There was no significant difference between the control mice (PBS treated) and MβCD or G15 treated mice (data not shown). These data indicate that inhibition of GPER1 and lipid rafts reduced SLE serum-induced skin inflammation.

## Discussion

Our study demonstrates that there is a higher incidence of SLE and more severe skin inflammation induced by SLE serum in female mice. We showed that monocytes are important in the development of SLE serum-induced skin inflammation and estrogen may enhance the activity of monocytes and SLE serum-induced skin inflammation through the membrane receptor GPER1. We also showed that lipid rafts exert an important role in estrogen mediating effects through GPER1.

Although the pathogenesis of SLE remains unclear, several lines of evidence suggest that SLE may be caused by immunological abnormalities including loss of B cell tolerance, abnormal interactions between T and B cell signaling, hyperactivity of immune cells, production of pathogenic autoantibodies, and defective clearance of auto-antigens and immune complexes (1, 31). The factor enhancing these abnormalities may promote pathological progression of SLE.

The observed female prevalence is highest after puberty. In (NZB × NZW) F1 (NZB/w F1) mice, the disease is more serious and has an earlier onset and higher mortality rate in females (32). This evidence suggests that estrogen may be involved in the onset and development of SLE. In this study, we examined 398 SLE patients and found that the female-to-male ratio was 15.5:1 and that 279 patients (83.7%) were in their reproductive age (18–45 years). Female mice had more serious skin lesions induced by SLE serum than males. Our data indicate that estrogen may play an important role in the development of SLE serum-induced skin inflammation.

We have previously reported that monocytes play a crucial role in the development of SLE serum-induced skin inflammation and that SLE serum IgG can induce monocyte differentiation into DCs (26). In this study, we confirmed our previous results. In addition, we found out that SLE IgG increased the levels of signaling molecules of cell activation (p-NF-κB p65) and inflammatory cytokines (MCP-1 and TNF-α) produced in monocytes. And we also found that FcγRs, which are widely expressed on cells throughout the hematopoietic system (33), were expressed on monocytes and that FcγRI (CD64) was expressed in skin lesions induced by SLE serum.

It has been shown that estrogens could modulate lymphoid cell growth and differentiation, activation and proliferation, cytokine secretion, and antibody production (14). Estrogens have been demonstrated to promote the pathological progression of SLE by inhibiting activation-induced apoptosis of SLE T cells through downregulating the Fas-L expression (34) and enhancing autoantibody levels in autoimmune diseases (35). Estrogens can also stimulate the production of IL-1, IL-4, IL-6, and IL-10 in macrophages and T cells (36). Estrogen exerts its effects by binding two types of receptors: nuclear receptors and cell membrane receptors (16). After binding EREs and recruiting coregulatory and chromatin remodeling proteins, ERα or ERβ could regulate the transcription of target genes (18). GPER1 is an integral membrane estrogen receptor that can trigger rapid estrogen-responsive signaling independent of ERα and ERβ (19). Our study indicates that GPER1 also plays an important role in skin injury of SLE. E2-BSA, which specifically binds to GPER1, increased the activation of monocytes and the production of inflammatory cytokines induced by SLE IgG and these effects were inhibited by G15, a specific GPER1 antagonist. On the other hand, a specific antagonist of nuclear estrogen receptors (ICI 182,780) did not inhibit the effect of E2-BSA on expression of p-NF-κB p65 induced by SLE IgG. In addition, we found that E2-BSA augmented skin inflammation induced by SLE serum and blockade of GPER1 reduced skin inflammation induced by SLE serum. These results indicate that estrogen promotes the monocyte activation induced by SLE IgG and skin inflammation induced by SLE serum through the membrane receptor GPER1 but not nuclear receptors.

Systemic lupus erythematosus IgG exerts its effect by binding FcγR on cell membrane. Our study supports that GPER1 regulates the effect of FcγR through lipid rafts. Lipid rafts which contain various signaling and transport proteins are sphingolipid- and cholesterol-rich domains of the plasma membrane (27). It has been reported that lipid rafts regulate the pathogenesis of organ and tissue damage in lupus-prone mice (28), play an important role in mediating the transport of substrates across the plasma membrane (27, 37, 38). In this study, we found that SLE IgG triggered lipid raft aggregation in monocytes and MβCD could inhibit the clustering of lipid rafts and monocyte differentiation into DCs. MβCD also inhibited monocyte activation and inflammatory cytokine production induced by SLE IgG. In addition, we demonstrated that aggregated lipid rafts contained CD64 and GPER1.

Our study suggests that the membrane estrogen receptor GPER1 is involved in the pathological progression of SLE and that estrogen may promote skin injury introduced by SLE serum through membrane receptor GPER1. Furthermore, lipid rafts play an important regulatory role in the effect of estrogen on the pathological progression of SLE through GPER1.

## Ethics Statement

This study was carried out in accordance with the recommendations of “IACUC of Nanjing Medical University” with written informed consent from all subjects. All subjects gave written informed consent in accordance with the Declaration of Helsinki. The protocol was approved by the “IACUC of Nanjing Medical University.” For statements involving animal subjects, please use: this study was carried out in accordance with the recommendations of “IACUC of guidelines, Nanjing Medical University of committee.” The protocol was approved by the “Nanjing Medical University of committee.”

## Author Contributions

ZC, CX, and G-MD conceived the idea and designed the research. ZC, XL, WQ, XG, HL, XF, XF, and G-MD initiated the study and drafted the manuscript; ZC and CX conducted the experiments; ZC, XL, CX, and G-MD analyzed the results. All the authors reviewed and approved the manuscript.

## Conflict of Interest Statement

The authors declare that the research was conducted in the absence of any commercial or financial relationships that could be construed as a potential conflict of interest.

## Supplementary Material

Figure S1 Skin inflammation induced by PBS, healthy serum, and systemic lupus erythematosus (SLE) serum. Histopathological photomicrograph **(A)** and severity of skin inflammation **(B)** in female C57BL/6 mice treated with PBS, healthy serum, and SLE serum (100 µl) in female C57BL/6 mice (*n* = 5 per group). H&E, original magnification 20×. ***P* < 0.01.

Figure S2 Different effects of estrogen receptors on activation of NF-κB triggered by systemic lupus erythematosus (SLE) IgG. **(A)** Western blot detected activation of NF-κB in RAW cells treated with SLE IgG, SLE IgG + E2-BSA, SLE IgG + E2-BSA + ICI, SLE IgG + ICI, and ICI alone. ICI is a specific inhibitor of estrogen nuclear receptors. **(B)** Western blot detected activation of NF-κB in RAW cells treated with SLE IgG, E2, E2-BSA, SLE IgG + E2, SLE IgG + E2-BSA, G15, SLE IgG + G15, and SLE IgG + E2-BSA + G15. G15 is a specific inhibitor of estrogen membrane receptor G protein-coupled estrogen receptor 1. Band densities of p-NF-κB p65 were quantified with densitometric analysis using ImageJ and normalized to the negative control treated with medium.

Figure S3 Effect of estrogen membrane receptor G protein-coupled estrogen receptor 1 (GPER1) on skin inflammation induced by systemic lupus erythematosus (SLE) serum. Histopathological photomicrograph **(A)** and severity of skin inflammation **(B)** in female C57BL/6 mice with intradermal injection of SLE serum (25 µl) in the presence or absence of GPER1 activator (E2-BSA, 250 nM) (*n* = 8 per group) and intraperitoneal injection of E2-BSA (250 nM). H&E, original magnification 20×. **P* < 0.05.
