# Corrigendum: The Role of Estrogen Membrane Receptor (G Protein-Coupled Estrogen Receptor 1) in Skin Inflammation Induced by Systemic Lupus Erythematosus Serum IgG

**DOI:** 10.3389/fimmu.2018.01732

**Published:** 2018-07-31

**Authors:** Zhenming Cai, Changhao Xie, Wei Qiao, Xibin Fei, Xuanxuan Guo, Huicheng Liu, Xiaoyan Li, Xiang Fang, Guangqiong Xu, Hui Dou, Guo-Min Deng

**Affiliations:** ^1^Key Laboratory of Antibody Techniques of Ministry of Health, Nanjing Medical University, Nanjing, China; ^2^State Key Laboratory of Reproductive Medicine, Nanjing Medical University, Nanjing, China; ^3^First Affiliated Hospital, Nanjing Medical University, Nanjing, China

**Keywords:** estrogen, systemic lupus erythematosus, IgG, skin inflammation, G protein-coupled estrogen receptor 1

Yacong Guo was included as an author in the published article. At his request, this author is being removed, and this does not change the scientific conclusions of the article in any way.

The original article has been updated.

## Conflict of Interest Statement

The authors declare that the research was conducted in the absence of any commercial or financial relationships that could be construed as a potential conflict of interest.

