# Supplementary figures and images for: The Role of Estrogen Membrane Receptor (G Protein-Coupled Estrogen Receptor 1) in Skin Inflammation Induced by Systemic Lupus Erythematosus Serum IgG
Source: Front Immunol. 2017 Dec 4;8:1723. doi: 10.3389/fimmu.2017.01723 (PMC5722986; doi:10.3389/fimmu.2017.01723)

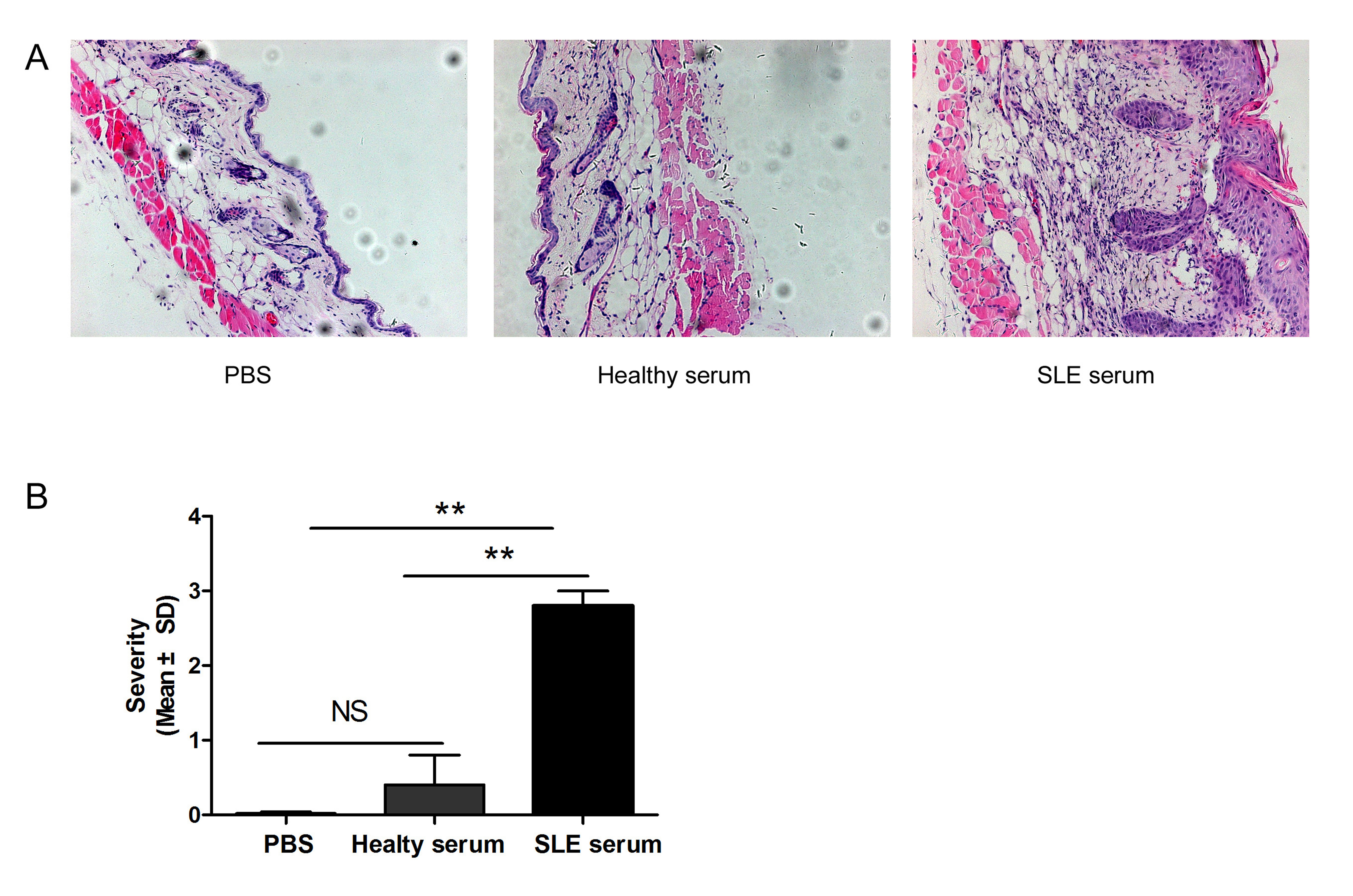

Supplement: Figure S1 — Skin inflammation induced by PBS, healthy serum, and systemic lupus erythematosus (SLE) serum. Histopathological photomicrograph (A) and severity of skin inflammation (B) in female C57BL/6 mice treated with PBS, healthy serum, and SLE serum (100 µl) in female C57BL/6 mice (n = 5 per group). H&E, original magnification 20×. **P < 0.01. [file Image_1.jpg]

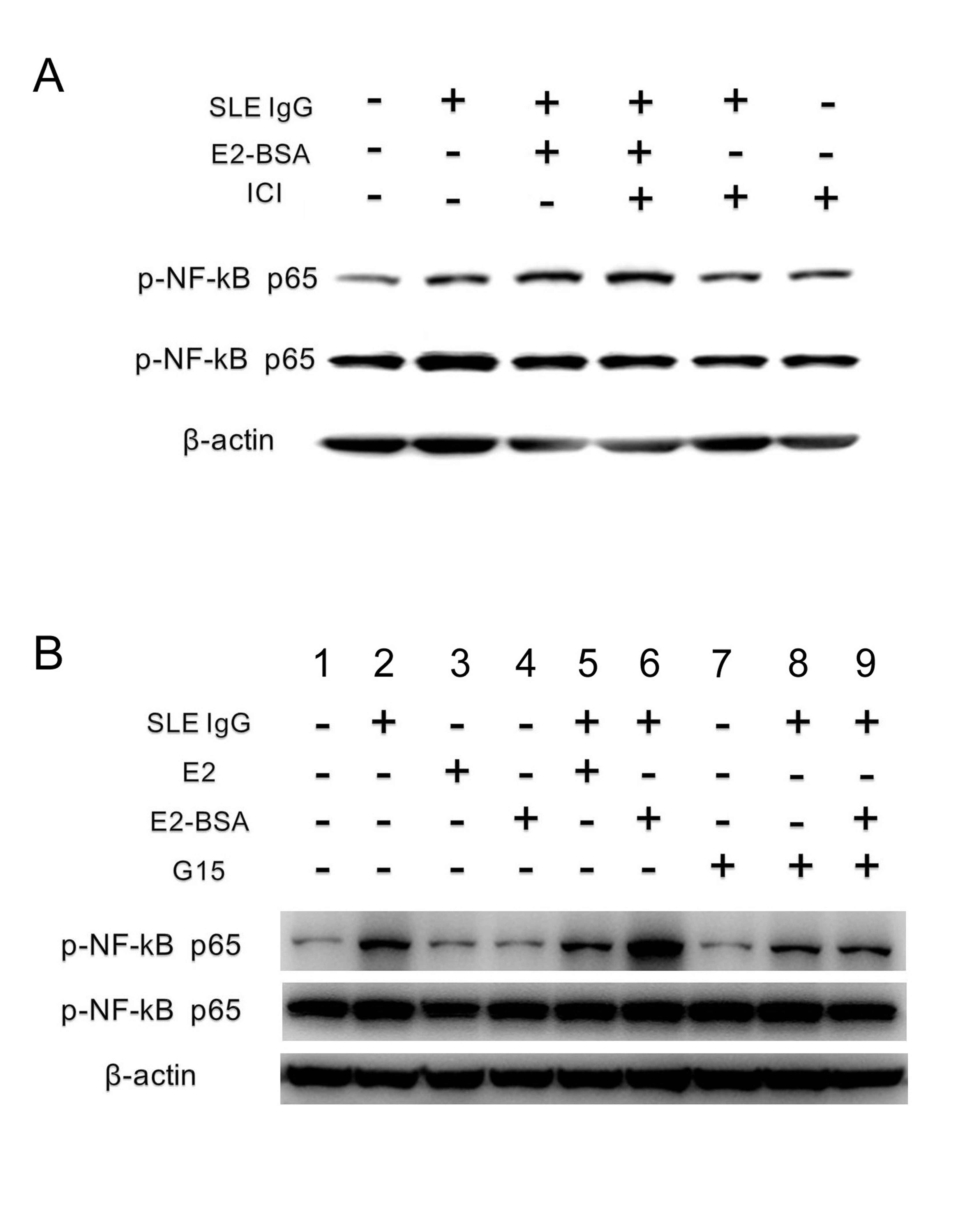

Supplement: Figure S2 — Different effects of estrogen receptors on activation of NF-κB triggered by systemic lupus erythematosus (SLE) IgG. (A) Western blot detected activation of NF-κB in RAW cells treated with SLE IgG, SLE IgG + E2-BSA, SLE IgG + E2-BSA + ICI, SLE IgG + ICI, and ICI alone. ICI is a specific inhibitor of estrogen nuclear receptors. (B) Western blot detected activation of NF-κB in RAW cells treated with SLE IgG, E2, E2-BSA, SLE IgG + E2, SLE IgG + E2-BSA, G15, SLE IgG + G15, and SLE IgG + E2-BSA + G15. G15 is a specific inhibitor of estrogen membrane receptor G protein-coupled estrogen receptor 1. Band densities of p-NF-κB p65 were quantified with densitometric analysis using ImageJ and normalized to the negative control treated with medium. [file Image_2.jpg]

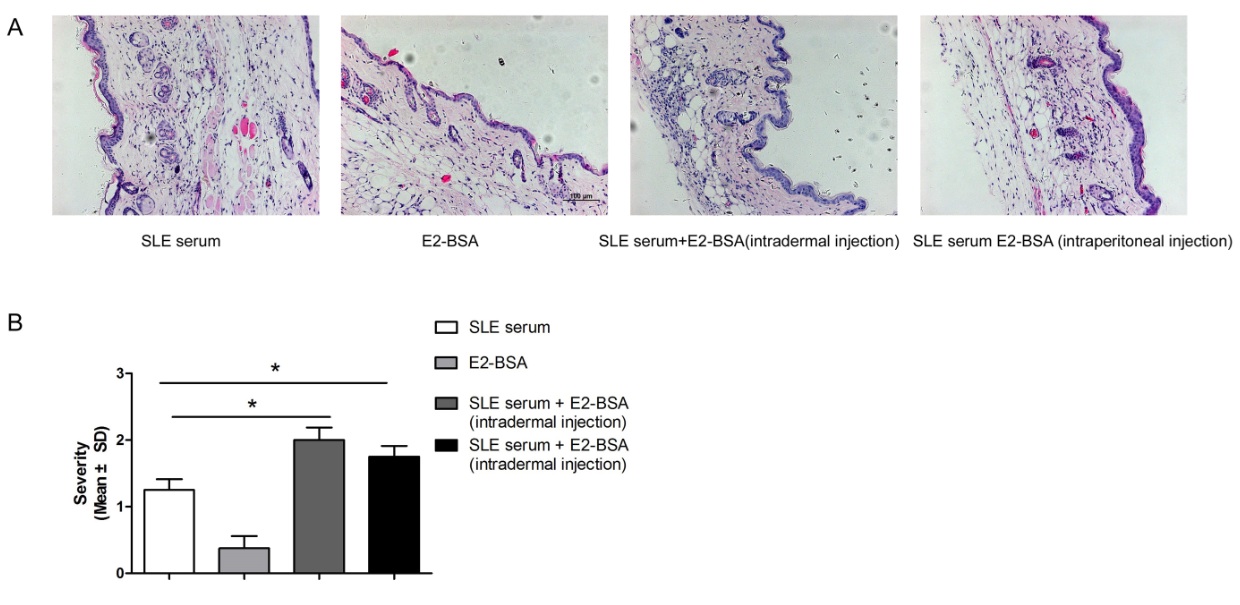

Supplement: Figure S3 — Effect of estrogen membrane receptor G protein-coupled estrogen receptor 1 (GPER1) on skin inflammation induced by systemic lupus erythematosus (SLE) serum. Histopathological photomicrograph (A) and severity of skin inflammation (B) in female C57BL/6 mice with intradermal injection of SLE serum (25 µl) in the presence or absence of GPER1 activator (E2-BSA, 250 nM) (n = 8 per group) and intraperitoneal injection of E2-BSA (250 nM). H&E, original magnification 20×. *P < 0.05. [file Image_3.jpg]
